# Prognostic Tools for Predicting Mortality in Older Adults With Hip Fracture: A Systematic Review

**DOI:** 10.7759/cureus.98351

**Published:** 2025-12-02

**Authors:** Gemma Badosa-Collell, Estel Call-Alsina, Jordi Amblàs-Novellas, Joan Carles Trullàs

**Affiliations:** 1 Department of Intermediate Care, Hospital d'Olot i Comarcal de la Garrotxa, Olot, ESP; 2 Tissue Repair and Regeneration Laboratory (TR2Lab), Institute for Research and Innovation in Life Sciences and Health in Central Catalonia (IRIS-CC) University of Vic - Central University of Catalonia, Vic, ESP; 3 Primary Care Team of Banyoles, Catalan Health Institute, Girona, ESP; 4 Multiprofessional Teaching Unit of Family and Community Care, Catalan Health Institute, Girona, ESP; 5 Central Catalonia Chronicity Research Group (C3RG), University of Vic - Central University of Catalonia, Vic, ESP; 6 Department of Internal Medicine, Hospital d'Olot i Comarcal de la Garrotxa, Olot, ESP; 7 Faculty of Medicine, University of Vic - Central University of Catalonia, Vic, ESP

**Keywords:** hip fracture, mortality, predictive model, predictive tool, roc curve, survival

## Abstract

Hip fracture (HF) is highly prevalent in older adults and is associated with functional impairment, disability, institutionalisation, increased use of healthcare resources, and mortality. Knowing the prognostic tools and identifying a gold standard to unify criteria would allow for more personalised clinical decision-making. The aim of this study is to conduct a systematic review of prognostic tools and their ability to predict mortality in patients over 65 years of age with HF. A search was conducted in the PubMed, Scopus, and Web of Science databases for studies published up to June 2022. The eligibility criteria were single-centre or multicentre studies published in English or Spanish that assessed the predictive ability of a prognostic tool for long-term mortality in patients over 65 years of age with HF. We identified 24 eligible publications. Most were single-centre observational cohort studies; 13 were prospective. The prognostic tools that showed better predictive capacity for 30-day mortality were the ASA score, age, cognitive status, gender, and Charlson Comorbidity Index score (ASAgeCoGeCC score) and the Almelo Hip Fracture Score (age, gender, haemoglobin, cognitive status, comorbidities, malignancy, mobility score, and ASA score), both with an area under the curve (AUC) of 0.82. The best predictive capacity for six-month mortality (AUC = 0.83) was reported for a nomogram that included age; albumin, sodium, and haemoglobin values; and Charlson Comorbidity Index score. The Frail-VIG index (including five domains: clinical, nutritional, functional, cognitive, and social) had the highest predictive capacity for one-year mortality (AUC = 0.90). There is great variability in tools for predicting mortality in patients with HF. Those that have greater predictive capacity include a multidimensional assessment.

## Introduction and background

Hip fracture (HF) is the main complication of osteoporosis and has a very high incidence in the older adult population [1]. It is associated with increased mortality, loss of function, decline in quality of life, and greater use of healthcare resources after discharge. The prevalence of HF is increasing due to population ageing, making it a matter of concern [1,2].

It is important to identify tools that can help differentiate between patients with a better or worse prognosis for survival at 12 months after an HF so as to improve outcomes. These tools are called predictive models or predictive tools. Early surgery has been shown to reduce the risk of mortality and postoperative complications. Furthermore, the preoperative assessment also presents an opportunity to stabilise patients, who often have multiple comorbidities. This can further prevent postoperative complications [3,4]. Reliable prognostic tools can be useful for effective preoperative preparation, which can subsequently reduce the risk of postoperative complications. These tools could also aid in determining the most suitable surgical and/or anaesthetic approach for each patient, thus optimising resources and improving the quality of patient care [4].

Numerous tools have been proposed in the literature to measure or predict mortality risk in order to improve the safety and well-being of older adult patients admitted with HF during the preoperative period. This study aims to conduct a systematic review of the literature to evaluate prognostic tools for mortality in patients over 65 years of age who present with an HF. It would be interesting to know if there is any tool that stands out above the others to serve as a gold standard.

## Review

Methods

A systematic review was conducted of published studies investigating prognostic tools and their ability to predict mortality in patients over 65 years of age with HF. The Preferred Reporting Items for Systematic Reviews and Meta-Analyses (PRISMA) guidelines [5] were followed during the conduct of this study, and the study protocol was registered in the PROSPERO international database of prospectively registered systematic reviews (registry number CRD42022340054) on 26 June 2022.

The inclusion criteria were: (1) single- or multicentre studies; (2) studies that evaluated a prognostic tool in patients with (any type of) HF; (3) studies that included patients older than 65 years; and (4) studies that evaluated short- or long-term mortality (one, six, and 12 months) as primary or secondary outcomes. The exclusion criteria were: (1) studies that did not provide primary data (i.e., review articles); (2) studies that included patients younger than 65 years; and (3) studies that only evaluated in-hospital mortality.

Studies were identified through a literature search in the PubMed, Scopus, and Web of Science (WOS) databases through 29 July 2022. The search terms “Hip fractures” and “Mortality” or “Survival” and “ROC curve” or “Predictive tool” or “Predictive model” were used. Only articles published in English or Spanish were included. The bibliographies of pertinent articles were also reviewed to identify additional studies.

Citations were uploaded to the Mendeley Desktop (version 1.19.8) reference manager software for title and abstract screening and data characterisation. Documents were reviewed based on title and abstract by two independent reviewers (GBC and ECA). Potentially eligible works were subsequently retrieved, and the full text was scrutinised for inclusion. A third investigator or “tiebreaker” (JCT) intervened in the event of disagreement. The data extracted included the first author’s name, year of publication, country (or countries) where the study was performed, number of patients included, mean age, gender, study design, name and description of the prognostic tool, and outcomes evaluated. An overview of the characteristics of eligible studies is shown in Table 1, and more details are provided in Table 2.

**Table 1:** Overview of the 24 articles included in the systematic review. ^a ^Countries: Europe: three studies each from Spain and the Netherlands; two each from Italy and Denmark; and one from Norway, Sweden, Austria, and the United Kingdom. Asia: four from China and two from South Korea. Americas: one each from the United States of America, Argentina, and Brazil. Oceania: one from Australia.

Characteristics of the publication	Number (Percentage)
Year of publication	
2013-2015	3 (12%)
2016-2018	5 (21%)
2019-2022	16 (67%)
Continent/countriesᵃ	
Europe	14 (58.3%)
Asia	6 (25%)
Americas	3 (12.5%)
Oceania	1 (4%)
Africa	0 (0%)
Study design	
Retrospective cohort studies	17 (71%)
Prospective cohort studies	7 (29%)
Sample size	
Median (range)	7,421 (75-55,716)
<500 patients	11 (46%)
501-999 patients	6 (25%)
>1000 patients	7 (29%)
Main outcomes	
30-day mortality	11 (46%)
6-month mortality	5 (21%)
12-month mortality	7 (29%)
Secondary outcomes	
6-month mortality	2 (8%)
12-month mortality	11 (46%)

**Table 2:** Characteristics of the 24 studies assessing prognostic tools for mortality in patients with hip fracture. AHFS: Almelo Hip Fracture Score; ACS-NSQIP: American College of Surgeons National Surgical Quality Improvement Program; ASA: American Society of Anesthesiologists physical status classification; AUC: area under the curve; BHFS: Brabant Hip Fracture Score; CCI: Charlson Comorbidity Index; CUPAX: Universal Questionnaire of Previous Extraordinary Activity; ECI: Elixhauser Comorbidity Index; F: female; FF: femur fracture; HGS: handgrip strength; Hip-MFS: Hip Multidimensional Frailty Score; HULP-HF: Hospital Universitario La Paz-Hip Fracture Score; IF-VIG: Frail-VIG index; mECM: modified Elixhauser Comorbidity Measure; NA: not available; NHFS: Nottingham Hip Fracture Score; Ortho-MPI: Orthopedic Multidimensional Prognostic Index; POSSUM: Physiological and Operative Severity Score for the Enumeration of Mortality and Morbidity. Primary outcomes are highlighted in shaded cells.

Author (Year, Country) [Ref]	Sample size / mean age / female	Cohort design	Prognostic tool(s)	30-day mortality*	6-month mortality*	12-month mortality*	Conclusions, limitations, risk of bias
Badosa-Collell G et al. (2021, Spain) [4]	N=103; 87 years; F: 76%	Retrospective	IF-VIG			AUC = 0.91	IF-VIG has good predictive capacity for mortality in older adults with FF. Limitation: only patients ≥85 years. Risk of bias: moderate.
Azevedo PS et al. (2017, Brazil) [6]	N=75; 80 years; F: 65%	Prospective	Goldman, Detsky, Lee		Goldman: OR 3.025 (1.022-8.953), p=0.046; Detsky: OR 2.328 (0.422-12.835), p=0.332; Lee: OR 1.262 (0.649-2.454), p=0.494		Only the Goldman score was associated with six-month mortality. Limitations: single-centre, small sample. Risk of bias: moderate.
Cenzer IS et al. (2016, USA) [7]	N=857; 84 years; F: 76%	Retrospective	Prognostic index			AUC = 0.72	The index differentiates low- vs high-risk one-year mortality after FF. Limitation: data collected within two years before FF, not immediately pre-event. Risk of bias: serious.
Choi JY et al. (2021, South Korea) [8]	N=242; 82 years; F: 73%	Retrospective	HGS, Hip-MFS, ASA		HGS: OR 1.101 (0.985-1.231), p=NS; Hip-MFS: OR 1.403 (1.027-1.917), p<0.005; ASA: OR 3.066 (1.106-8.503), p<0.005		Hip-MFS and ASA, but not HGS, predicted 6-month mortality. Limitations: single-centre, retrospective; postoperative complications were the main outcome. Risk of bias: serious.
Choi JY et al. (2017, South Korea) [9]	N=481; 80 years; F: 71%	Retrospective	Hip-MFS		AUC = 0.78; OR 1.419 (1.239-1.626), p<0.001		Hip-MFS predicts six-month mortality after FF. Limitation: single-centre, retrospective. Risk of bias: moderate.
Crespo-Fresno A et al. (2022, Spain) [10]	N=206; 87 years; F: 80%	Prospective	CUPAX	AUC = 0.69	AUC = 0.69		CUPAX is useful in older adults with FF. Limitation: single-centre. Risk of bias: moderate.
Dawe EJ et al. (2013, UK) [11]	N=259; 85 years; F: 78%	Prospective	Sernbo score	AUC = 0.71		AUC = 0.68	Simple tool for routine assessment to identify high-risk older adults pre-op. Limitation: only intracapsular FF. Risk of bias: low.
Fu G et al. (2020, China) [12]	N=702; 77 years; F: 77%	Retrospective	Nomogram			AUC = 0.76	Nomogram predicts one-year mortality and walking ability pre-op in FF slated for arthroplasty. Limitations: single-centre, retrospective. Risk of bias: serious.
Garabano G et al. (2021, Argentina) [13]	N=135; 87 years; NA	Retrospective	CCI			AUC = 0.70	CCI suitable for one-year mortality. Limitations: small sample; retrospective; valid only for ≥75 years with unstable intertrochanteric FF. Risk of bias: moderate.
Guo J et al. (2021, China) [14]	N=2,241; 79 years; F: 68%	Retrospective	Base model vs CCI, mECM, MFI	AUC = 0.76 / 0.76 / 0.77 / 0.76		AUC = 0.62 for all scores	Base model showed good discrimination for all-cause mortality. Limitations: single-centre, retrospective; largest AUC below “fair” threshold. Risk of bias: moderate.
Haugan K et al. (2021, Norway) [15]	N=3,651; 84 years; F: 70%	Retrospective	CCI vs ASA	AUC = 0.72 / 0.72		AUC = 0.75 / 0.73	CCI and ASA similar predictive capacity; ASA easier to use. Limitations: single-centre, retrospective. Risk of bias: low.
Hjelholt T et al. (2022, Denmark) [16]	N=28,791; 82 years; F: 70%	Retrospective	New tool			AUC = 0.74	Model is useful with information available at admission. Limitations: no cognitive assessment; retrospective. Risk of bias: low.
Lau TW et al. (2016, China) [17]	N=759; 84 years; F: 72%	Retrospective	CCI	AUC = 0.79		AUC = 0.71	CCI correlated well with mortality. Limitations: non-operated FF excluded; single-centre, retrospective. Risk of bias: serious.
Mellner C et al. (2021, Sweden) [18]	N=55,716; 83 years; F: 69%	Retrospective	Sernbo score			AUC = 0.69	Sernbo is easy to use and identifies high one-year mortality risk. Limitation: 30% missing data on cognitive impairment; retrospective. Risk of bias: moderate.
Menéndez-Colino R et al. (2020, Spain) [19]	N=509; 85 years; F: 79%	Prospective	HULP-HF			AUC = 0.79	HULP-HF slightly higher predictive capacity for one-year mortality vs prior studies. Limitation: no external validation. Risk of bias: low.
Nia A et al. (2021, Austria) [20]	N=1,101; 84 years; F: 70%	Retrospective	ACS-NSQIP vs POSSUM, P-POSSUM, CCI	AUC = 0.72 / 0.70 / 0.71 / 0.67	AUC = 0.72 / 0.69 / 0.69 / 0.70		ACS-NSQIP best of the four. Limitations: single-centre, retrospective. Risk of bias: moderate.
Nijmeijer WS et al. (2016, Netherlands) [21]	N=850; 83 years; F: 74%	Prospective	Almelo Hip Fracture Score vs NHFS	AUC = 0.82 / 0.72			AHFS more useful than NHFS for identifying frail older adults at high early-mortality risk after FF. Limitation: single-centre. Risk of bias: moderate.
Pan L et al. (2022, China) [22]	N=454; 82 years; F: 73%	Retrospective	Nomogram		AUC = 0.83	AUC = 0.79	New nomogram shows good accuracy/usefulness post-FF. Limitations: no external validation; single-centre, retrospective. Risk of bias: moderate.
Toson B et al. (2015, Australia) [23]	N=47,698; NA; F: 73%	Prospective	CCI	AUC = 0.72-0.75		AUC = 0.69-0.75	CCI predicts 30-day and one-year mortality after FF. Limitation: data from administrative database (retrospective in nature). Risk of bias: moderate.
Trevisan C et al. (2021, Italy) [24]	N=323; 84 years; F: 78%	Retrospective	ASAgeCoGeCC vs CCI, NHFS	AUC = 0.82 / 0.78 / 0.76		AUC = 0.81 / 0.76 / 0.80	ASAgeCoGeCC showed good calibration and excellent discrimination up to 4 years after FF. Limitations: single-centre, retrospective; needs external validation. Risk of bias: moderate.
van de Ree CL et al. (2019, Netherlands) [25]	N=993; 82 years; F: 70%	Retrospective	Brabant Hip Fracture Score	AUC = 0.71		AUC = 0.75	BHFS showed acceptable discrimination and adequate calibration for 30-day and one-year mortality. Limitation: retrospective. Risk of bias: moderate.
Vesterager JD et al. (2022, Denmark) [26]	N=31,443; NA; F: 69%	Prospective	Base model, CCI, ECI, Rx-Risk, combinations	AUC = 0.69 / 0.72 / 0.71 / 0.72		AUC = 0.67 / 0.71 / 0.71 / 0.71	Highest discrimination at 30 days and one year by combining CCI and Rx-Risk with age and gender. Risk of bias: moderate.
Vitale E et al. (2014, Italy) [27]	N=95; 84 years; NA	Retrospective	Ortho-MPI		OR 1.05 (1.004-1.11)		Ortho-MPI could predict outcomes in older adults with FF. Limitations: small sample; single-centre; very high 6-month mortality (75%). Risk of bias: serious.
Wesdorp MA et al. (2021, Netherlands) [28]	N=422; 84 years; F: 75%	Retrospective	Almelo Hip Fracture Score	AUC = 0.70			AHFS useful and easy to use for predicting 30-day mortality after FF surgery. Limitations: single-centre; some variables collected retrospectively. Risk of bias: moderate.

With regard to ethics, this study complies with the Declaration of Helsinki. Ethical approval and consent to participate were not applicable due to this study’s nature as a systematic literature review.

Results

Study Identification

**Figure 1:**
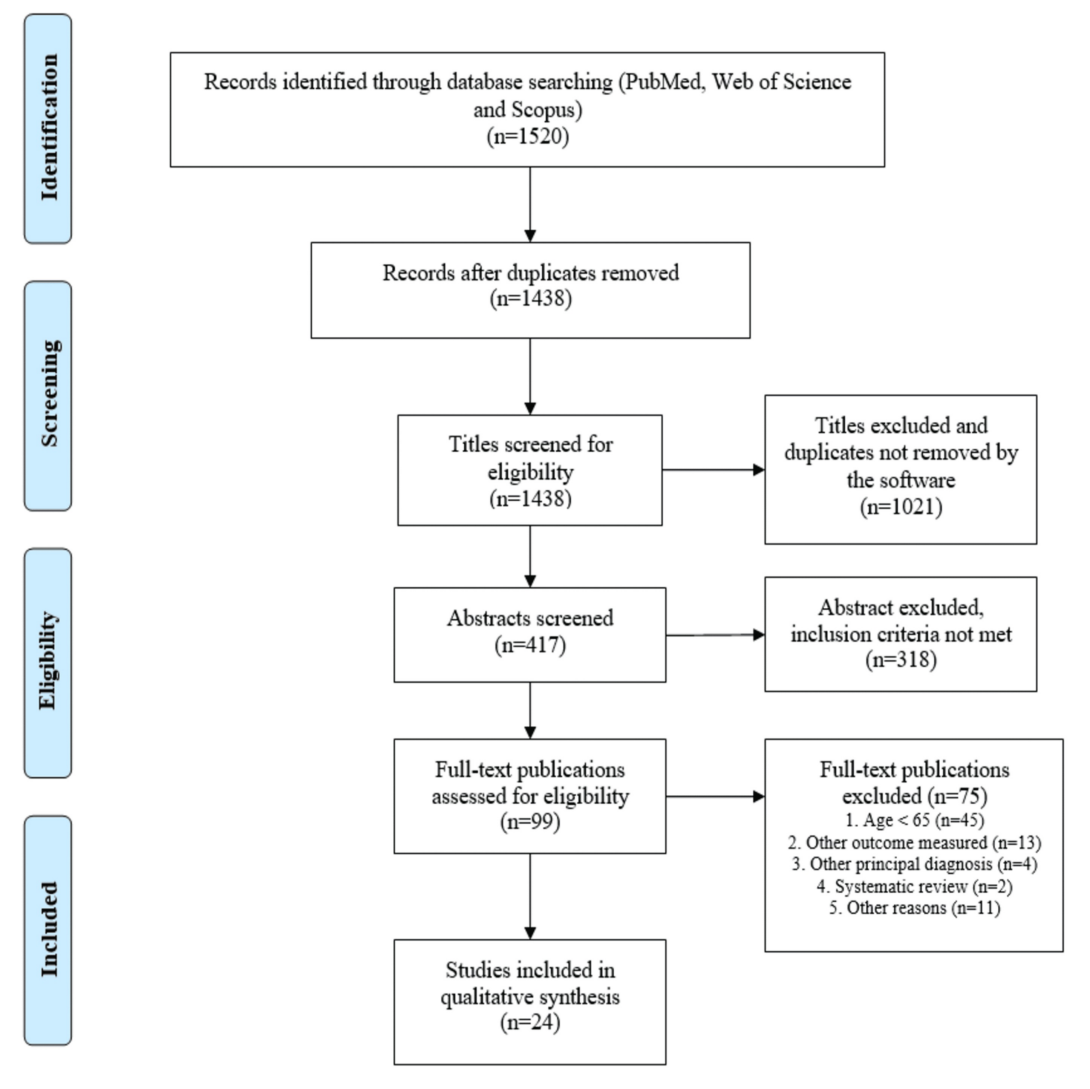
PRISMA flow diagram showing the stages of the review and the numbers of records identified, screened, excluded, and included. PRISMA: Preferred Reporting Items for Systematic Reviews and Meta-Analyses.

A total of 1,520 potentially relevant publications were initially identified in the three databases (PubMed, Scopus, and WOS), as shown in the PRISMA flow diagram in Figure 1. After the initial screening, 1,339 articles were excluded based on title and abstract. The remaining 99 potentially relevant publications were identified for further review and, after examining these articles in greater detail, 24 were ultimately included in the systematic review.

Study Characteristics

The main characteristics of the 24 studies selected are overviewed in Table 1 and detailed in Table 2. The studies were published between 2013 and 2022, and all were observational cohort studies (only seven were prospective and the remaining 17 were retrospective). They were mainly conducted in Europe. The sample sizes ranged from 75 to 55,716 patients. Overall, the mean age of the patients included in the studies was 83 years, and there was greater representation of females (70% on average). The outcome most frequently evaluated was 30-day mortality (11 studies), followed by 12- and 6-month mortality (seven and five studies, respectively). Since the results obtained in the studies are very heterogeneous, they do not allow us to conduct an in-depth subgroup meta-analysis.

Risk of Bias Assessment of Included Studies

All studies included in this review are cohort studies; therefore, we evaluated the risk of bias using the ROBINS-I scale. The seven domains of bias assessed were: confounding, patient selection, classification of interventions, deviations from intended interventions, missing data, measurement of outcomes, and selection of reported results. Overall, most studies presented a moderate risk of bias, as detailed in Table 2, particularly in the domains of patient selection (especially retrospective studies) and missing data. According to the GRADE system, the overall risk of bias was considered moderate.

Prognostic Tools for Mortality

The 24 studies included in this systematic review assessed the prognostic value of 28 prognostic tools. The characteristics and variables or domains they include are shown in Table 3. Briefly, the prognostic tools are very heterogeneous but usually share some common variables or domains [4,6-27]. Almost all the prognostic tools evaluate age and comorbidities (in 20 and 24 cases, respectively), followed by cognitive or emotional status in 15 cases, gender and analytical parameters in 13 cases, and functional status in 12. Lastly, seven prognostic tools also included an evaluation of geriatric syndromes [4,6-27].

**Table 3:** Prognostic tools and their variables or dimensions. ACS-NSQIP: American College of Surgeons National Surgical Quality Improvement Program; ASA: American Society of Anesthesiologists physical status classification; CCI: Charlson Comorbidity Index; CUPAX: Universal Questionnaire of Previous Extraordinary Activity; ECI: Elixhauser Comorbidity Index; ECG: electrocardiogram; Hip-MFS: Hip Multidimensional Frailty Score; HULP-HF: Hospital Universitario La Paz-Hip Fracture Score; IF-VIG: Frail-VIG index; mECM: modified Elixhauser Comorbidity Measure; Na⁺: sodium; NHFS: Nottingham Hip Fracture Score; Ortho-MPI: Orthopedic Multidimensional Prognostic Index; POSSUM: Physiological and Operative Severity Score for the Enumeration of Mortality and Morbidity; Ref: references. The variables or dimensions evaluated are highlighted in shaded cells.

Prognostic tool^ref.^	Age	Gender	Comorbidities	Geriatric syndromes	Cognitive and/or emotional variables	Functional status	Analytical parameters	Other variables
IF-VIG [4]			√	√	√	√		Socioeconomic data
Goldman score [6]	√		√					ECG
Detsky score [6]	√		√					
Lee score [6]	√		√					
Prognostic Index [7]	√	√	√	√		√		Socioeconomic data
Hip-MFS [8,9]		√	√	√	√	√	Serum albumin	
Handgrip Strength [9]								
CUPAX [10]						√		
Sernbo score [11,18]	√				√	√		Socioeconomic data
Nomogram [12]	√		√		√	√	Serum albumin	ECG and chest X-ray
CCI [13-15,17,20,23,26]	√		√					
Base model [14]	√	√	√				Haemoglobin	Type of fracture
Multidimensional frailty [14]			√	√	√	√	Serum albumin	
ASA [9,15]			√					
New tool [16]	√	√	√	√				Institutionalisation; type of fracture
HULP-HF [19]	√	√	√		√	√	D vitamin and haemoglobin	
ACS-NSQIP [20]	√	√	√			√		
POSSUM [20]	√		√		√		Blood count and biochemistry	Type of fracture; ECG and chest X-ray
p-POSSUM [20]	√	√	√		√		Blood count and biochemistry	Type of fracture; ECG and chest X-ray
NHFS [21,24]	√	√	√		√		Haemoglobin	Institutionalisation
Almelo Hip Fracture Score [21,28]	√	√	√		√	√	Haemoglobin	Institutionalisation
Nomogram [22]	√		√				Serum albumin, Na+ and haemoglobin	
ASAgeCoGeCC [24]	√	√	√		√			
Brabant Hip Fracture Score [25]	√	√	√		√		Haemoglobin	Institutionalisation
Base model [26]	√	√						
Rx-Risk [26]			√	√	√	√		
mECM and ECI [14,26]			√		√		Haemoglobin	
Ortho-MPI [27]	√	√	√	√	√	√	Haemoglobin	

Results for the 30-Day Mortality Outcome

**Figure 2:**
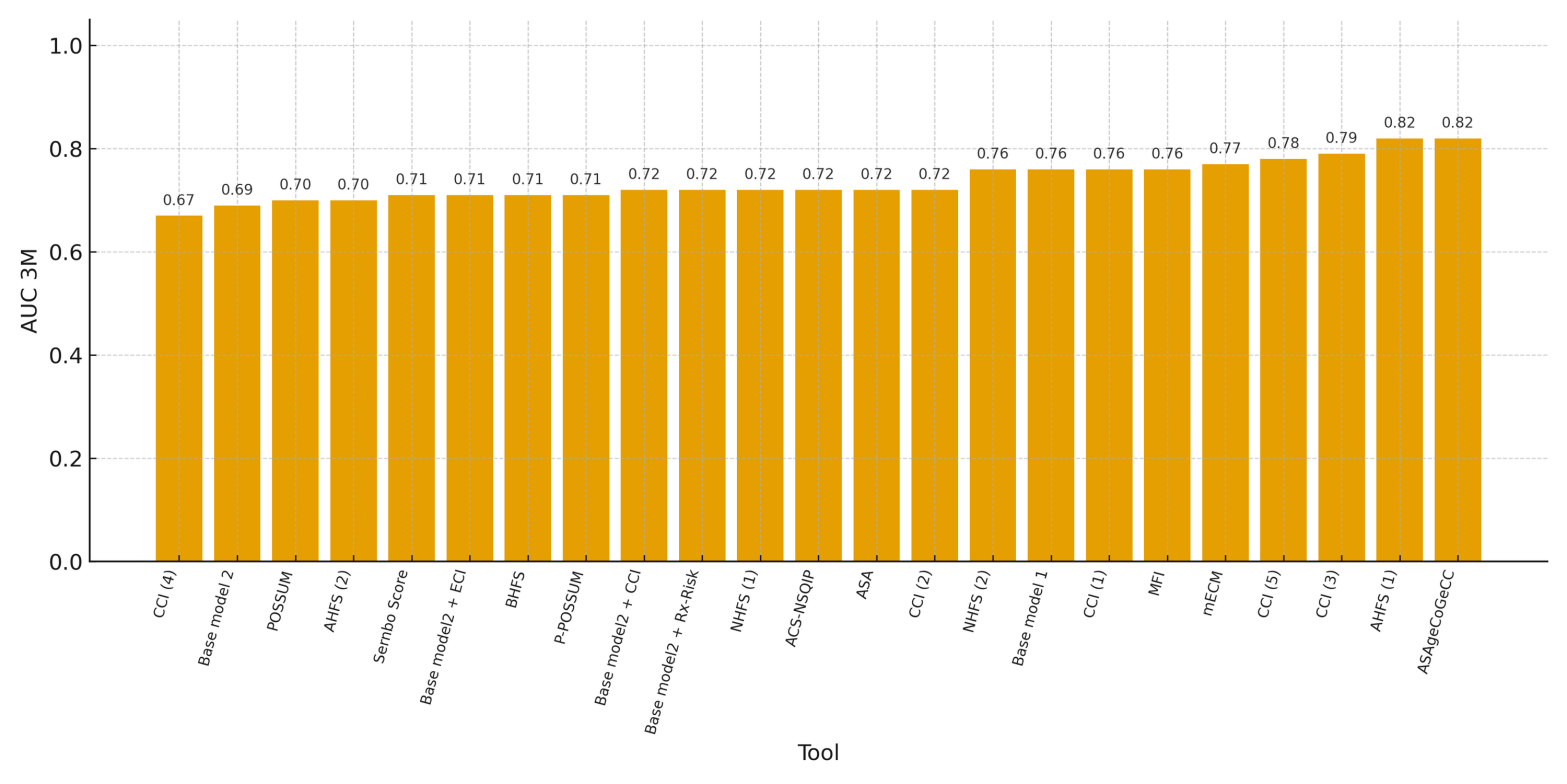
3-month mortality prognostic tools. AHFS: Almelo Hip Fracture Score; ACS-NSQIP: American College of Surgeons National Surgical Quality Improvement Program; ASA: American Society of Anesthesiologists physical status classification; AUC: area under the curve; BHFS: Brabant Hip Fracture Score; CCI: Charlson Comorbidity Index; ECI: Elixhauser Comorbidity Index; mECM: modified Elixhauser Comorbidity Measure; NHFS: Nottingham Hip Fracture Score; POSSUM: Physiological and Operative Severity Score for the Enumeration of Mortality and Morbidity. Base model 1 corresponds to the tool described in reference [14], and Base model 2 corresponds to the tool described in reference [26]. Three tools have been assessed and compared in more than one study, showing different AUC values: CCI1 [14], CCI2 [15], CCI3 [17], CCI4 [20], CCI5 [24]; AHFS1 [21], AHFS2 [28]; NHFS1 [21], NHFS2 [24].

Eleven studies evaluated 30-day mortality as the main outcome. As shown in Tables 2-3, the prognostic tools used to predict 30-day mortality were very heterogeneous but frequently entail evaluation of the following variables or domains: age, gender, comorbidities, cognitive and/or emotional variables, and functional status. The prognostic tools with better predictive capacity (measured by area under the curve (AUC)) for 30-day mortality were the ASAgeCoGeCC score (including the ASA score, age, gender, cognitive status, and Charlson Comorbidity Index (CCI)) [24] and the Almelo Hip Fracture Score (including age, gender, comorbidities, cognitive and functional status, haemoglobin values, and institutionalisation) [21], both with an AUC of 0.82. Figure 2 describes the predictive capacity of all the 30-day mortality prognostic tools.

Results for the Six-Month Mortality Outcome

**Figure 3:**
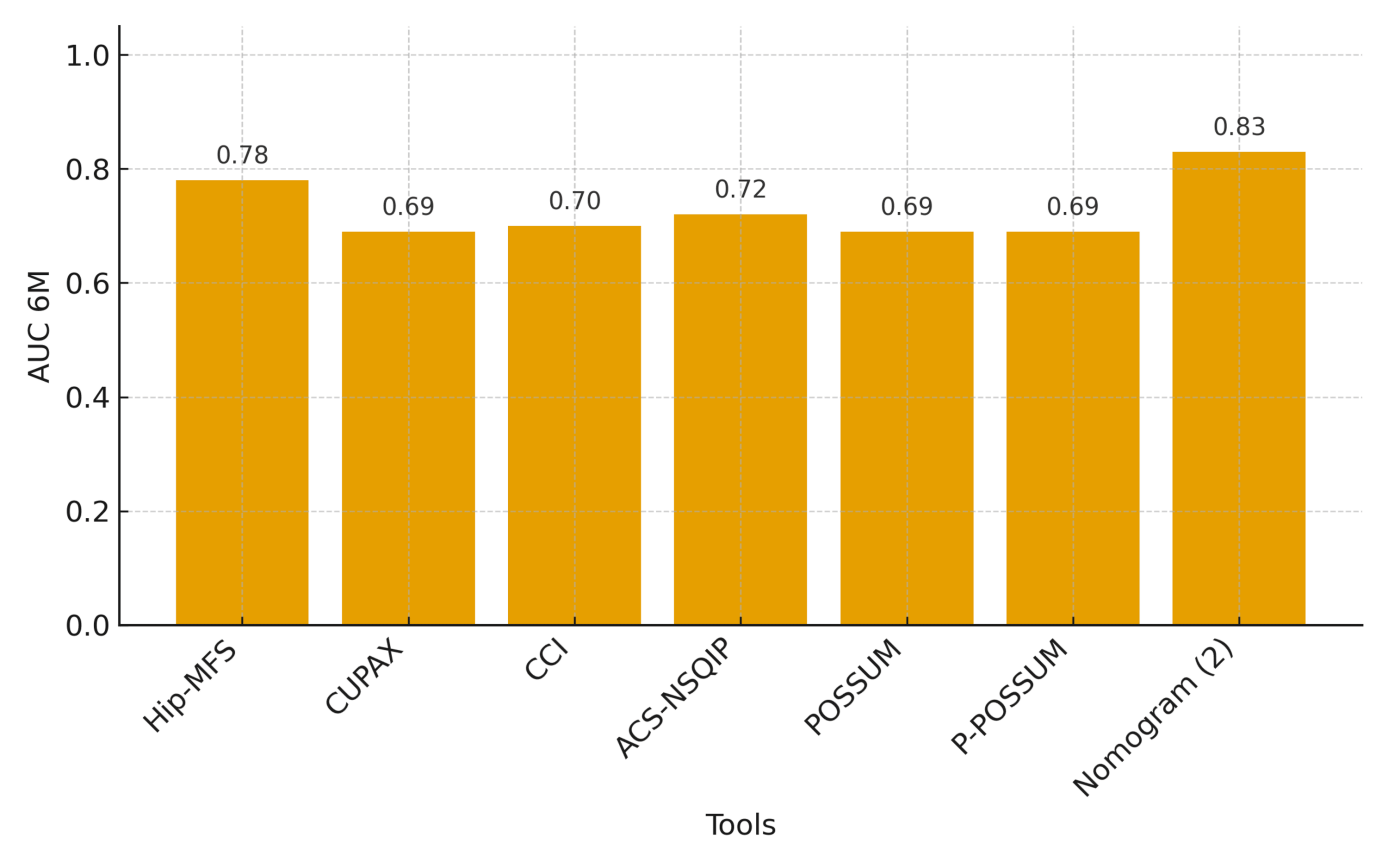
6-month mortality prognostic tools. ACS-NSQIP: American College of Surgeons National Surgical Quality Improvement Program; AUC: area under the curve; CCI: Charlson Comorbidity Index; CUPAX: Universal Questionnaire of Previous Extraordinary Activity; Hip-MFS: Hip Multidimensional Frailty Score; POSSUM: Physiological and Operative Severity Score for the Enumeration of Mortality and Morbidity. Nomogram 2 corresponds to reference [22].

Six-month mortality was evaluated as the main outcome in five studies and as a secondary outcome in two studies. The prognostic tools used to evaluate six-month mortality were also heterogeneous and are detailed in Tables 2-3. The tool that showed the best predictive capacity was a nomogram that included age, comorbidities, and analytical parameters (including haemoglobin, sodium, and albumin), with an AUC of 0.83 [22]. Figure 3 describes the predictive capacity of all the 6-month mortality prognostic tools.

Results for the 12-Month Mortality Outcome

**Figure 4:**
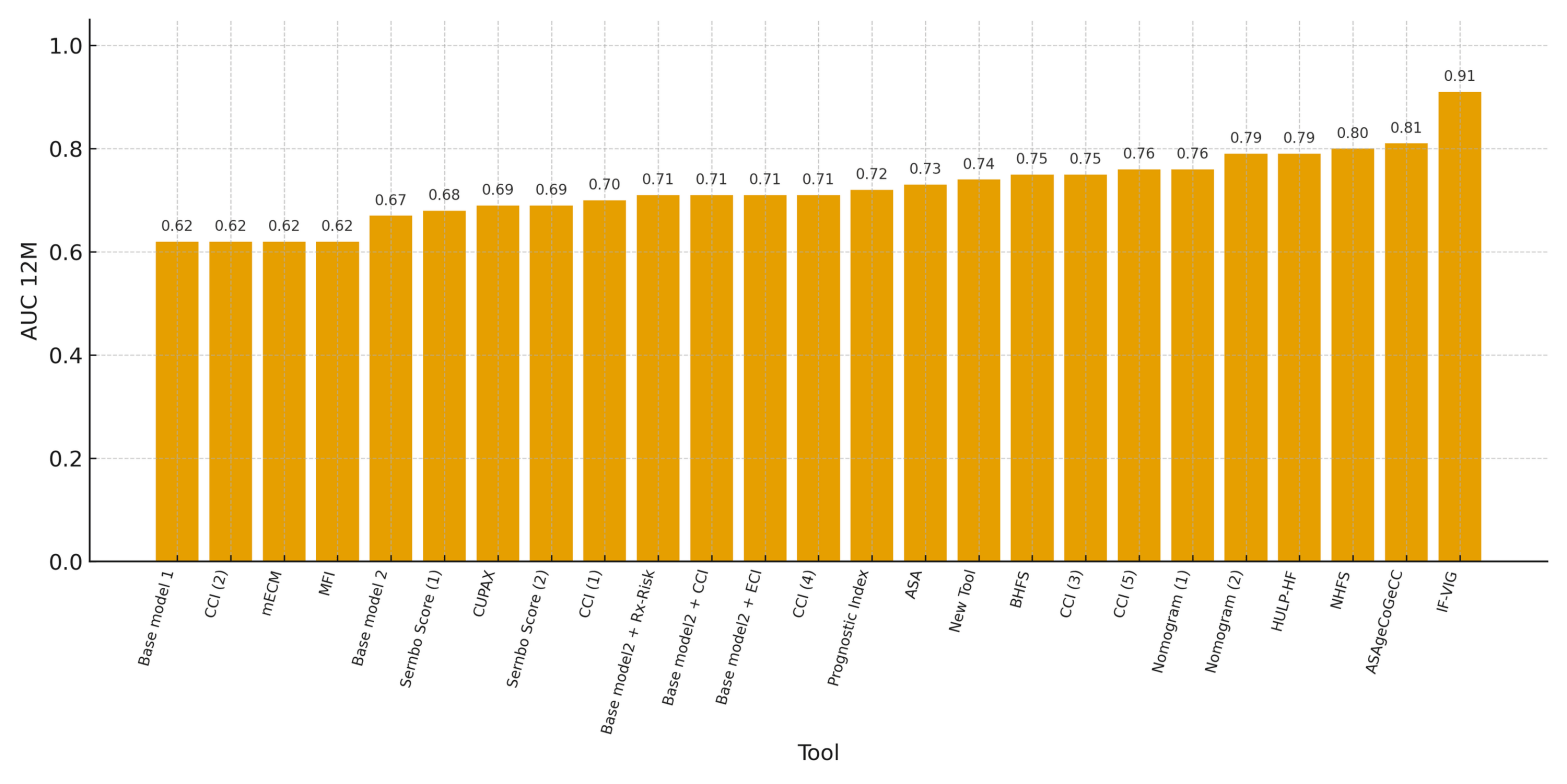
12-month mortality prognostic tools. ASA: American Society of Anesthesiologists physical status classification; AUC: area under the curve; BHFS: Brabant Hip Fracture Score; CCI: Charlson Comorbidity Index; CUPAX: Universal Questionnaire of Previous Extraordinary Activity; ECI: Elixhauser Comorbidity Index; Hip-MFS: Hip Multidimensional Frailty Score; HULP-HF: Hospital Universitario La Paz-Hip Fracture Score; IF-VIG: Frail-VIG index; mECM: modified Elixhauser Comorbidity Measure; NHFS: Nottingham Hip Fracture Score; POSSUM: Physiological and Operative Severity Score for the Enumeration of Mortality and Morbidity. Base model 1 corresponds to reference [14]; Base model 2 corresponds to reference [26]; Nomogram 1 corresponds to reference [12]; Nomogram 2 corresponds to reference [22]. Two tools have been assessed and compared in more than one study, showing different AUC values: Sernbo score 1 [11], Sernbo score 2 [18]; CCI1 [13], CCI2 [14], CCI3 [15], CCI4 [17], CCI5 [24].

Lastly, eighteen studies evaluated 12-month mortality: seven as the primary outcome and eleven as a secondary outcome. As with the other outcomes, the tools used to evaluate 12-month mortality are heterogeneous but share common variables or dimensions (Tables 2-3). The Frail-VIG index [4] (which includes 22 variables from five domains: clinical, nutritional, functional, cognitive, and social) had the highest predictive capacity for one-year mortality, with an AUC of 0.91. Figure 4 describes the predictive capacity of all the 12-month mortality prognostic tools.

Discussion

The results of this systematic review show great variability in the prognostic tools used to predict mortality after an HF. Despite this heterogeneity, most prognostic tools evaluate similar variables or domains, the most common being age, comorbidities, cognitive and/or emotional status, and functional status. It should be noted that most of these studies have been carried out in Europe, possibly because there is a large older adult population and femur fracture is prevalent [29,30].

Most studies evaluated short-term mortality (at 30 days), with predictive capacity (according to the AUC) ranging from 0.64 to 0.82. The tools with the highest predictive capacity (AUC of 0.82) were the Almelo Hip Fracture Score and ASAgeCoGeCC, both of which include variables and domains that are easy to collect in clinical practice (age, gender, comorbidities, and cognitive/emotional status) [21,24]. It should be noted that there are two studies in our review that validate the Almelo tool. The Wesdorp MA et al. study shows an AUC of 0.70. Comparing the two studies, both have substantial sample sizes, although the sample in the Wesdorp MA et al. study is smaller. Additionally, in the original Almelo population [21], the high-risk group is more institutionalised and presents greater cognitive impairment than the high-risk population in the Wesdorp MA et al. study [28].

Fewer studies assessed six-month mortality using the area under the curve. A nomogram developed by Pan L et al. showed the highest predictive capacity for six-month mortality, with an AUC of 0.83. Again, this nomogram evaluates age and comorbidities as well as analytical parameters that are easy to measure in clinical practice [22].

Finally, regarding one-year mortality, the Frail-VIG index was the prognostic tool that demonstrated the highest predictive capacity, with an AUC of 0.91. The Frail-VIG index includes 22 variables from five domains (clinical, nutritional, functional, cognitive, and social) that are also easy to obtain in clinical practice [4,31].

It is striking that the Nottingham Hip Fracture Score (NHFS), a well-known and widely used score to predict mortality in patients with HF in different European countries, was not included in this systematic review [32,33]. The NHFS was not included because the studies conducted for its development and validation were carried out in patients under 65 years of age, which was an exclusion criterion for this systematic review [32,33]. However, we believe it is a useful tool. In fact, our group has published a study, after carrying out this review, demonstrating its utility in the geriatric population with hip fracture [34].

There are some limitations to this study that should be considered. First, this review included 24 articles out of the 1,520 initially identified. Despite efforts to be as comprehensive as possible, some articles may have been missed. To avoid this, the reference lists of the eligible articles were also reviewed to detect possible publications not found in the initial search. Second, this systematic review has the inherent limitations of the studies included: most studies were single-centre, retrospective cohort studies, and the prospective studies usually had small sample sizes. To increase the level of evidence of these prognostic tools, they should be tested in randomised controlled trials. Also, only published studies were included; thus, publication bias may have affected the results. Finally, most of the studies were carried out in Europe and Asia, so the results cannot be extrapolated to other continents.

In summary, this systematic review found that different tools have been tested to predict mortality after an HF. Most evaluate similar domains that are easy to obtain in clinical practice, and they usually show good predictive capacity. Despite this, it is necessary to conduct more prospective studies to assess the predictive capacity of these tools in larger populations.

## Conclusions

There is a considerable number of studies evaluating mortality prediction in patients with hip fracture; however, most are retrospective and have small sample sizes. Despite this, we observed considerable variability in tools for predicting mortality in older adults with hip fracture. Those with greater predictive capacity are multidimensional assessments that take into account age, comorbidities, and cognitive and functional status.
